# Older Adults’ Experiences With Participation and eHealth in Care Coordination: Qualitative Interview Study in a Primary Care Setting

**DOI:** 10.2196/47550

**Published:** 2023-10-02

**Authors:** Hilde Marie Hunsbedt Fjellså, Anne Marie Lunde Husebø, Harald Braut, Aslaug Mikkelsen, Marianne Storm

**Affiliations:** 1 Department of Public Health Faculty of Health Sciences University of Stavanger Stavanger Norway; 2 Research Group of Nursing and Health Sciences Stavanger University Hospital Stavanger Norway; 3 Department of Innovation, Leadership, and Marketing Business School University of Stavanger Stavanger Norway; 4 Stavanger University Hospital Stavanger Norway; 5 Faculty of Health Sciences and Social Care Molde University College Molde Norway

**Keywords:** care coordination, older adults, participation, eHealth, primary health care

## Abstract

**Background:**

Owing to the demographic changes in the elderly population worldwide, delivering coordinated care at home to multimorbid older adults is of great importance. Older adults living with multiple chronic conditions need information to manage and coordinate their care. eHealth can be effective for gaining sufficient information, communicating, and self-managing chronic conditions. However, incorporating older adults’ health preferences and ensuring active involvement remain challenging. More knowledge is needed to ensure successful participation and eHealth use in care coordination.

**Objective:**

This study aimed to explore multimorbid older adults’ experiences with participation and eHealth in care coordination with general practitioners (GPs) and district nurses (DNs).

**Methods:**

The study had a qualitative explorative approach. Data collection included semistructured interviews with 20 older adults with multimorbidity receiving primary care services from their GPs and DNs. The participants were included by their GPs or nurses at a local intermunicipal acute inpatient care unit. The data analysis was guided by systematic text condensation.

**Results:**

We identified 2 categories: (1) older adults in charge of and using eHealth in care coordination, and (2) older adults with a loss of control in care coordination. The first category describes how communication with GPs and DNs can facilitate participation, the importance of managing own medication, and how eHealth can support older adults’ information needs. The second category focuses on older adults who depend on guidance and help from their GPs and DNs to manage their health, describing how a lack of capacity and system support to be involved makes these adults lose control of their care coordination.

**Conclusions:**

Being in charge of care coordination is important for older multimorbid adults. The results show that older adults are willing to use eHealth to be informed and to seek information, which ensures high levels of participation in care coordination. Future research should investigate how older adults can be involved in electronic information sharing with health care providers.

## Introduction

Older adults with multimorbidity (co-occurrence of two or more chronic conditions) [1] often experience challenges with care coordination and navigating the health care system [2-5]. Demographic changes in the elderly population worldwide have led to a necessity to treat and care for older adults with chronic conditions at home [6-8]. To meet these challenges, engaging older adults in managing their health and navigating health care services is essential and can reduce health care use [9-11]. Care coordination can be defined as the deliberate organization of patient care activities between two or more participants (including the patient) involved in a patient’s care to facilitate the appropriate delivery of health care services. Organizing care involves the marshaling of personnel and other resources needed to carry out all required patient care activities and is often managed by the exchange of information among participants responsible for different aspects of care [3]. McDonald et al [3] emphasized that the patient’s perspective is essential to care coordination. It is the patients who evaluate whether the coordination of care is experienced as sufficient [12]. The care coordination measurement framework describes different measures that illustrate what mechanisms meet the patient’s needs and the delivery of high-quality health care services. Key care coordination mechanisms described by McDonald et al [3] are communication among involved personnel, information sharing, facilitating transition, supporting patients’ self-management goals, and assessing patients’ needs and goals. Other broader approaches to care coordination are medication management and health information technology–enabled coordination. These broader approaches to care coordination can facilitate the overall improvement of health care service delivery [3].

Health care professionals and older adults with chronic illnesses highlight the importance of participating in health-related decision-making and achieving self-management goals [13,14]. Receiving health-related information and managing one’s health and care are important health-promoting activities that enable patient participation [14-16]. The Word Health Organization (WHO) defines participation as “being involved in a life situation” [17]. Thompson [16] describes 5 levels of patient involvement, from the lowest to the highest: (0) Noninvolvement: patients can lack medical knowledge, have low self-confidence, and receive care as passive patients; (1) Information seeking or information receptive: patients are provided information; (2) Information giving or dialogue: patients are listened to and heard, and patients and professionals share information; (3) Shared decision-making: patients make informed choices, receive guidance, and express their opinions in cooperation with professionals; and (4) Autonomous decision-making: patients can make choices independently, manage their health, and decide for themselves if they want to inform the professionals. Patients describe participation in managing their health as feeling respected, being heard, and being listened to by health care professionals who acknowledge their perspectives on their health situation [18].

A key purpose of participation is to provide relevant information to the patients according to their individual needs [18]. Despite a wide variation in the amount of health information needed by multimorbid older adults [19], many desire access to information about their health and some use eHealth to do so [4]. eHealth is defined as “the use of information and communication technologies (ICT) for health” [20]. A recent scoping review identified the following eHealth tools used in care coordination for older adults: patient portals, electronic health journals, telehealth monitoring solutions (sensor technology, virtual ward, and video consultations), and the use of the telephone [21]. These tools can ensure information sharing, enable communication between older adults and health care professionals, and support self-management of chronic illnesses [3,21-23].

eHealth can be an enabler for older adults to take an active role in their care [21,24,25]. Scholz Mellum et al [26] described how older adults with multimorbidity coordinate their care as they desire to be in control of their health. However, Elliot et al [27] reported that older adults living at home do not participate frequently in decisions regarding their health. Lack of time and difficulties in sharing and receiving information about patients were reported by the study participants (primary care nurses, social workers, and care coordinators) [27]. A review by Peart et al [28] showed that health care professionals assisted the self-management of home-dwelling older adults by actively involving them in care planning. The patients appreciated that their needs were respected by health care professionals [28].

Knowledge, confidence, and support from health care professionals and family members are factors that can enable the participation of home-living older adults in coordinating their care and their use of eHealth [21]. However, when supporting older adults’ needs, health care professionals have found it challenging to incorporate older adults’ health preferences [27,29]. Care coordination programs or interventions alone are insufficient to ensure the reduced use of health care [9]. The lack of incorporation of older adults’ health preferences and the insufficiency of care coordination programs call for other approaches to ensure successful participation and eHealth use in care coordination. Therefore, this study aimed to explore multimorbid older adults’ experiences with participation and eHealth in care coordination with general practitioners (GPs) and district nurses (DNs) [27]. The following research question guided our study: How do older adults experience participation in care coordination with GPs and DNs, and how does eHealth support their participation? This knowledge can allow primary health care services to develop strategies for delivering services that engage older adults, promote the self-management of chronic illnesses, and ensure the successful use of eHealth in care coordination [10,11,30].

## Methods

### Study Design

This was an explorative qualitative study conducted on health care services in a Norwegian municipality. Norwegian health care services are based on principles, such as universal coverage and equal access to health care services for all. The health care services are organized at a specialist and primary care level [31]. Norway, along with other Scandinavian countries, ranks the highest in digitalization. For example, all Norwegian citizens must use electronic identification to access banking services [32]. In 2017, they were introduced to a personalized patient portal with the following features: an overview of medication prescriptions, appointments at the hospital or with GPs, access to hospital medical record systems, and the possibility of sending electronic messages to their GPs [33]. Primary care services are responsible for prevention, treatment, and early diagnostics. In addition, primary care services have financial responsibilities for citizens when hospitals consider them ready to be discharged to their municipalities [34,35]. Norwegian municipalities deliver home care services to 3.6% (203,000) of the residents in Norway, and the number of older adults receiving services is increasing [36]. Norwegian home care services can assist and educate community-dwelling patients in performing practical chores, such as cleaning the house, taking out the garbage, and cleaning clothes. Other services provided by home care services are health-related services in the home, such as district nursing services or providing day services for older adults to socialize, share meals, and be active [36]. All residents in Norway have the right to a GP. GPs are employed at different GP offices within the municipality, which are open during the day from Monday to Friday [37]. In addition, municipalities are responsible for emergency services, such as local emergency rooms that are open 24 hours a day, 7 days a week. Citizens in a municipality who need acute treatment are either referred to the local intermunicipal acute inpatient care (AIC) unit or the nearby regional hospital [37].

### Study Setting and Participants

The study was conducted in a Norwegian municipality with 80,000 residents, where older adults live both in rural and urban areas. The district nursing services are organized into 5 different districts. The municipality has about 70 GPs who are co-located in different GP offices. The GP offices have nurses and administrative health personnel responsible for the telephone and for conducting clinical tests (eg, blood pressure examinations, blood tests, and spirometry).

We used convenience sampling to recruit older adult participants, and they were recruited either by their GPs or by a nurse working at the AIC unit [38,39]. The research team (HMHF and HB) reached out to each of the GP offices in the municipality and invited the GPs located there to participate in the study. We used the following inclusion criteria when recruiting the older adults: age >65 years, having two or more medical conditions, being on four or more medications, living at home, receiving district nursing, and having a hospital admission within the last 12 months before inclusion in the study. The GP or nurse at the AIC unit assessed if the included participants met the inclusion criteria. For older adults to be included in the study, all inclusion criteria had to be met. A total of 24 older adults were invited to participate, and 20 older adults were included. The reasons for declining to participate were not wanting the interview to be audio recorded and not finding the research relevant.

### Data Collection

We used a semistructured interview guide developed by the research team (HMHF, HB, AM, and MS). The team consisted of researchers with competencies in social science, leadership, eHealth, general medicine, or nursing. The interviewed adults were asked about their age, gender, and medical conditions; the reason for hospital admission; what services they received from the municipality; and who followed their postadmission care. The interview guide focused on the following aspects related to experiences with care coordination: methods of communication with their GPs and DNs, use of eHealth, participation in decisions regarding their health, and efforts by health care professionals to involve them in coordination. We ensured that the older adults were given questions that were open-ended and that they provided examples of what they did in a situation (eg, what they did to manage medications or how they ensured that they understood the information given by health care professionals about their health or services). The interviews lasted between 30 and 90 minutes, and were carried out between October 2019 and February 2020. The first and third authors (HMHF and HB) conducted the interviews and had weekly meetings during the data collection period. The older adults were interviewed in their homes or at the AIC unit.

### Ethical Considerations

This study is part of the research project “Leadership and Technology for Integrated Health Care Services” reviewed and registered with the Norwegian Agency for Shared Services in Education and Research (Sikt) (reference number: 228630). Sikt ensures data protection and legal access to handle research data. The research project was exempt from a formal review by the Regional Committee for Medical and Health Research Ethics in Norway (reference number: 2019/1138) because the project did not intend to generate further knowledge about health and disease. The research team (HMHF and HB) contacted the older adults after they provided oral consent to their GPs or the leader at the AIC unit to participate in the study. All participants were provided with written and oral information about the study. They were informed about the aims, methods, potential risks, and benefits of the research project according to the Helsinki Declaration [40]. Participants were informed that participation was voluntary and that they had the right to withdraw from the study at any time without giving any reason. The data were collected and stored according to data protection regulations.

### Data Analysis

The interviews were audio recorded and transcribed verbatim. All personal and identifying information was removed to ensure the anonymity and confidentiality of the interview participants. NVivo 12 [41] was used to analyze the text. The analysis was guided by the 4-stage systematic text condensation approach described by Malterud [42] to present the data material as condensed text in categories with subcategories.

#### Stage 1

HMHF, HB, AM, and MS read all the data material and gained a general overview of the data associated with older adults’ experiences with participation and eHealth in care coordination with GPs and DNs. This resulted in the identification of the following preliminary themes: communication with health care professionals, experiences with the use of eHealth, information flow, and medication management. All the authors met in an analysis meeting to discuss the data material and preliminary themes.

#### Stage 2

In the second stage of the analysis, units of meaning from the transcribed material were identified, and code groups based on the preliminary themes were established. HMHF coded the data and had several analysis meetings with MS and AMLH, who provided input on the codes and progression of the data analysis.

#### Stage 3

The code groups were grouped into 7 subgroups and reviewed. HMHF made a condensate illustrating each subgroup. The condensate was a summary of the original transcribed data material.

#### Stage 4

The subgroups were divided into 2 categories. We synthesized the condensate to text describing the categories and subgroups, and illustrated the text with quotes from the participants. Table 1 provides examples of the analysis process, with extracts from the data material.

**Table 1:** Examples of the systematic text condensation analysis process.

Preliminary theme	Meaning unit (quote)	Subgroup	Category
Medication management	The DNs had control of my medication before. But there were so many errors in the medication from a dispenser that I stopped the service. Firstly, some medications are missed. Then there was some medication I did not recognize. Then, I said, let me do it myself, administrate the medication. I take the medication in the evening and morning. [75-year-old woman]	Managing own medication Excerpt from condensation of the subgroup: I am in control of my medications; I trust myself more than others. Even though I receive medication on a roll, I manage some medication myself. I adjust the dosage on my own. I have done that for many years, so I know what I am doing	Older adults in charge of and using eHealth in care coordination
Experiences with the use of eHealth	The GP has all information on the computer and can monitor it. They can bring out my whole history from the hospital. I feel confident that they know why I am there and will control my blood values and so on. [84-year-old man]	Using eHealth to be informed or to seek health information Excerpt from condensation of the subgroup: The GPs and DNs communicate electronically, but they tell me if there is some information that’s about me. I know that the hospital gives the GP electronic reports. This information is important to me and my GP. It gives me confidence that the GPs can access my electronic journal, especially if I am going to another doctor who is not my GP.	Older adults in charge of and using eHealth in care coordination

In the final stage of our analytic work we used a deductive approach. Thompson's 5 levels of patient involvement [16] were used to systematize the results to illustrate participation in care cordination among multimorbid older adults.

## Results

### Description of the Participants and eHealth Use

The mean age of the study participants was 82 years (range, 71-98 years). Of the 20 participants, 13 (65%) were female and 7 (35%) were male. As described in Table 2, the participants had various health problems, including chronic obstructive pulmonary disease, heart failure, chronic pain, cancer, physical disability, depression, and anxiety. A majority of the older adults in the study used the telephone to contact their GPs or home care nurses. Some had safety alarms, sent electronic messages to their GPs, or used electronic patient portals to share and access information about their health, such as appointments and prescriptions for their medications. A few interviewees used ICT, played games on their iPads or tablets, or paid their bills using their computers. Some had help from their caregivers to send text messages or call GPs or DNs. One participant was blind and was not able to access the electronic patient portal.

**Table 2:** Overview of participants’ gender, age, self-reported health problems, and eHealth use.

Participant gender and age	Health problems	eHealth use
Woman, 82 years	COPD^a^, heart failure, and chronic pain	Uses the telephone to call GPs^b^ or DNs^c^ Has a safety alarm
Woman, 98 years	Cancer and reduced mobility	Uses the telephone to call GPs, DNs, or other health care providers Has a safety alarm
Man, 71 years	Physical disability	Uses the telephone and a tablet Uses an electronic patient portal to control prescriptions and appointments, and to access the medical journal Uses the telephone to call GPs Receives text messages about appointments at the GP office or hospital
Man, 88 years	Chronic pain	Uses the telephone to call GPs or DNs Receives information from GPs via text messages Has a safety alarm
Man, 84 years	Impaired eyesight	Uses the telephone to call GPs or DNs Uses a tablet to log into the electronic health portal Makes appointments with GPs electronically
Woman, 81 years	COPD	Uses the telephone to call GPs or DNs Has a safety alarm
Man, 84 years	Blindness, diabetes, and physical disability	Uses the iPhone and Siri functions to write text messages and call GPs Has a safety alarm
Woman, 85 years	COPD, heart condition, diabetes, and femur fracture	Uses the telephone to call GPs or DNs Has a safety alarm
Woman, 85 years	Hypertension, metabolic syndrome, cancer wound, and femoral neck fracture	Uses the telephone to call GPs or DNs Has a safety alarm
Woman, 71 years	COPD, heart failure, depression, and chronic pain	Uses the telephone, tablet, and computer Has a safety alarm Uses the computer to write online consultations with GPs Uses Google to find information about treatments
Man, 80 years	No report of illness or health problems^d^	Uses the telephone to call GPs or DNs
Man, 88 years	No report of illness or health problems^d^	Uses the telephone to call the GP or DNs Has a safety alarm
Woman, 75 years	Digestive issues and insulin use due to unstable blood sugar	Uses the telephone to call GPs Has a safety alarm
Woman, 87 years	No report of illness or health problems^d^	Uses the telephone to call GPs Next of kin helps with calling DNs Has a safety alarm
Man, 80 years	No report of illness or health problems^d^	Uses the telephone to call GPs GPs can send text messages, including information on appointments Has a safety alarm
Woman, 84 years	Anxiety and issues with pneumonia	Uses the telephone to call GPs or DNs
Woman, 90 years	Chronic pain and bone fragility	Uses the telephone to call GPs Next of kin helps send text messages to GPs
Woman, 72 years	COPD, paralysis due to cerebral stroke, and cerebral vision impairment	Uses Google to get health information Uses the telephone to call GPs or DNs, but often receives assistance from the next of kin Has a safety alarm
Woman, 88 years	Hypertension, anxiety, and urinary tract issues	Uses the telephone to call GPs or DNs Next of kin helps send text messages to GPs Has a safety alarm
Woman, 82 years	Chronic pain and cerebral stroke	Uses the telephone to call GPs Sends text messages to GPs

^a^COPD: chronic obstructive pulmonary disease.

^b^GP: general practitioner.

^c^DN: district nurse.

^d^Did not self-report health problems.

### Older Adults in Charge of and Using eHealth in Care Coordination

The ability to communicate with GPs and DNs was central for the older adults to be in charge of coordinating their care. Managing their medications and using eHealth to be informed or to seek health information were aspects of engagement in care coordination.

#### Communicating With GPs and DNs

Communicating with GPs or DNs was essential for the older adults to be in charge of care coordination. To experience useful collaboration, they had to be able to talk to GPs and DNs, express their thoughts, and ask questions. Several interviewees talked about how they experienced collaboration with GPs as useful and how they could talk about their thoughts regarding their health. Furthermore, GPs educated them on health issues or treatments. Several participants had GPs who asked them about their opinions related to the treatment or follow-up of their health issues. A few of the older adults said that their GPs inquired about any other services and referred them to the health and welfare office or the hospital, if necessary. Many interviewees stated that they wanted to participate in and oversee their health. One older adult described that he was in control of his life and stated what the GP should do to help him:

I have taken the lead in my own life. I am in charge, not the GP or someone else or you. I am in charge and that’s it! (...) That is how I want it to be to the bitter end. Because it is not the GP that should take the lead in the patient's life. It is the patients that live it themselves. (…) You [the GPs] should just provide the patient with tools.

88-year-old man

Many participants had monthly appointments with their GPs, which they booked in advance. This gave them an arena to communicate with their GPs and ask questions about issues concerning their medical condition or about the information they did not understand. The appointments with GPs were also important for giving assurance of follow-up on various tests. Some appreciated the opportunity to talk to their GPs as they had many health issues.

Sometimes the GP uses some expressions, and then I ask “What do you mean by that?” The GP could have said “today your blood values are fine”, and your blood pressure is so and so. Instead of me having to ask and ask.

81-year-old woman

Several interviewees had daily visits by DNs, while others had weekly visits. Some personnel were nurse assistants, while others were registered nurses, responsible for tasks such as administering medication and injections. DNs assisted some of the older adults to get in contact with GPs, and also shared information with GPs about their medical conditions, medications, or health care appointments. Patients also used DNs for medical advice when they felt ill. DNs guided the older adults on what to do and performed measurements, such as blood pressure or blood sugar assessment, as illustrated by a participant:

I have asked the DNs if they can come and test my blood sugar since the blood sugar sometimes has too high levels.

85-year-old woman

DNs were described as being nice. They were people to talk to about daily life and were always available for support. A few participants perceived DNs as having experience with caring for patients with reduced mobility and having knowledge on how to help them. Some interviewees perceived that DNs were updated on their situation and could inform them about new medications:

Yes, the DNs will always stop by the day I arrive home [after being transferred home from the hospital]. If there are changes in my medication, the DNs are updated and inform me about the new medication.

82-year-old woman

#### Managing Own Medications

Managing medications was an important aspect of older adults’ involvement in care coordination. Many of the older adults participated in dispensing their medications from multidose dispensers, which informed them of the date and time to take the medications. Some also had other medications on the side that they managed themselves, sometimes in dialogue with their GPs and sometimes without involving their GPs. A few took paracetamol (acetaminophen) when they had pain and wanted to manage the pain medication themselves. One of the female participants explained that she discussed her medications with the medical doctors at the AIC unit and that they had decided that it was safe for her to be in charge of the pain medications herself.

A few interviewees explained that they trusted themselves more than health care professionals when it came to taking the correct medication and dosage at the right time. In addition, they received the information they needed from the pharmacy about side effects, and when and how to take the prescribed medication. According to these interviewees, this information was not given by DNs or GPs. Some participants experienced changes in their medication after being discharged from the hospital, or when they received a multidose medication dispenser. They remembered what medication they were supposed to take, and in that way, they made sure that the medication was as prescribed. One woman explained that she found errors in the multidose medication dispenser and that she stopped the service and took control of the medication management herself:

The DNs had control of my medication before. But there were so many errors in the medication from a dispenser that I stopped the service. Firstly, some medications are missed. Then there was some medication I did not recognize. Then I said, let me do it myself, administrate the medication. I take the medication in the evening and morning.

75-year-old woman

#### Use of eHealth to be Informed or to Seek Health Information

Access to eHealth ensured that older adults could participate in care coordination by obtaining or seeking information over the telephone, patient portals, or the internet. Although all the interview participants had access to their patient portals, they rarely took advantage of this opportunity. Most of the older adults included in the study were able to use the telephone to contact their GPs, DNs, or the hospital. A few interviewees said they did not like to use the telephone to call GPs or DNs, but they were able to use the telephone to contact their family members or friends. Some explained that if there were test results or some health-related information they did not understand or needed more information about, they could call the GP office and ask questions. Even though they did not reach the GPs when they called, the nurses knew who they were and ensured that they got an answer from the GPs. Some interviewees said they did not feel the need to read their medical records or log into their patient portals, as they preferred to talk to their GPs or DNs face-to-face. A few did not know they could access the electronic journal from the hospital through a patient portal.

Do we have that (online access to their electronic journal)? I have not tried to get hold of that information (the electronic journal), and I have not had the need. I have been informed enough as it is kind of. I can ask if there is something I wonder about.

82-year-old woman

A few older adults used the patient portal to be updated on appointments or medication prescriptions, or to send electronic messages to their GPs. They pointed out that they felt secure knowing that their GPs could always access the information about them, as information from the hospital is transferred electronically to their GPs.

The GP has all information on the computer and can monitor it. They can bring out my whole history from the hospital. I feel confident that they know why I am there and will control my blood values and so on.

84-year-old man

Some interviewees explained that their GPs called them to inform them about test results, even though that happened rarely. Only a few participants used the internet to gain information about their health conditions and find possible treatments that they suggested to their GPs.

Yes, she (the GP) listens to me. The last time I was there with heart failure, I Googled and then I read something that perhaps it can be surgically treated. Something like a pacemaker or a heart starter. Yes, for heart failure it said.

71-year-old woman

One interviewee pointed out that older people need to be taught how to use ICT, making sure that older adults can be updated on available eHealth services.

People are getting older and older, and policymakers cannot expect people to use everything on the computer and digitally. They must teach the older group too… so that people are updated.

90-year-old woman

### Older Adults With a Loss of Control in Care Coordination

Being dependent on guidance from GPs and DNs, and lacking the capacity to be involved in information sharing were aspects resulting in a loss of control in care coordination.

#### Dependence on Guidance and Help From GPs and DNs in Managing Health

Some of the interviewees experienced challenges that limited their participation in care coordination. Some participants lacked health literacy and needed help managing their medications. Several of the older adults talked about respecting and trusting the GP’s knowledge. They perceived that the GP had the best knowledge about their health problems and how to treat them properly. Some explained that they needed help from GPs and DNs to manage their medical conditions. Receiving health care services was necessary to manage their health. Therefore, they followed the instructions and plans of the GPs on how to self-manage their medical conditions. Seemingly, some older adults lacked sufficient health literacy to know what is best for them and had to trust GPs:

I don’t know what is best for me. It is the GP that suggests different solutions. I follow the GPs advice without thinking more about it. I trust the GPs assessment.

84-year-old man

Some of the interviewees needed more help managing their medications. DNs helped some patients to remember to take their medications and signed on a sheet of paper when they had taken the medication. Other participants had reduced physical function, and some explained that GPs or DNs had to assist them in administering eye drops or taking medications at the right time. One female participant also said that she wanted to put the medication in the dispenser herself, but she had difficulties with her fine motor skills and needed assistance from DNs.

In the beginning, I put the medication in the medication dispenser myself. I do not do that anymore. It is a long time ago. I miss it because I like to be in control.

90-year-old woman

#### Lack of Capacity and System Support to be Involved in Care Coordination

Some older adults could not remember relevant health information and experienced difficulty becoming involved in care coordination. A lack of system support also hampered participation. Many of the interviewees perceived that they did not remember all information regarding their health after being discharged home from the hospital or AIC ward, or after meeting their GPs. They were too sick to be able to remember all information. A few interviewees experienced the information sharing with DNs as challenging. Having several DNs or nurse assistants coming to their homes made it hard to get to know those working in the district nursing service. Therefore, some participants did not share important information, such as information on recent hospital admissions or hospital discharge letters, because of a lack of continuity with DNs.

I don’t talk with the DNs. I don’t even mention that I have been hospitalized. I think they know, but I can’t relate to them as there always is a new DN stopping by. If there had been one permanent nurse, I could at least talk with them about stuff, but no, it is not like that. They just give me my medications and say goodbye.

81-year-old woman

A few participants described a lack of collaboration with their GPs due to the infrequency of visits to the GP office. They said that GPs lacked information about their current health status, as illustrated by a female participant:

I think there has been very little collaboration with the GP. He called me, and then I had an appointment with him right after I was discharged home from the AIC unit. So, I have visited the GPs office, but it seems he does not have a full understanding of what has happened to me. He kind of ask me things I thought he should know.

72-year-old woman

Some older adults experienced not being involved in electronic information sharing between DNs and GPs. They did not know what was written in the electronic messages exchanged between DNs and GPs. One participant said:

I don´t like it. They can ask me. They can come to me and ask me directly and talk to me before they write something (in the journal). But they don´t do that.

90-year-old woman

### Participation in Care Coordination Among Multimorbid Older Adults

**Figure 1:**
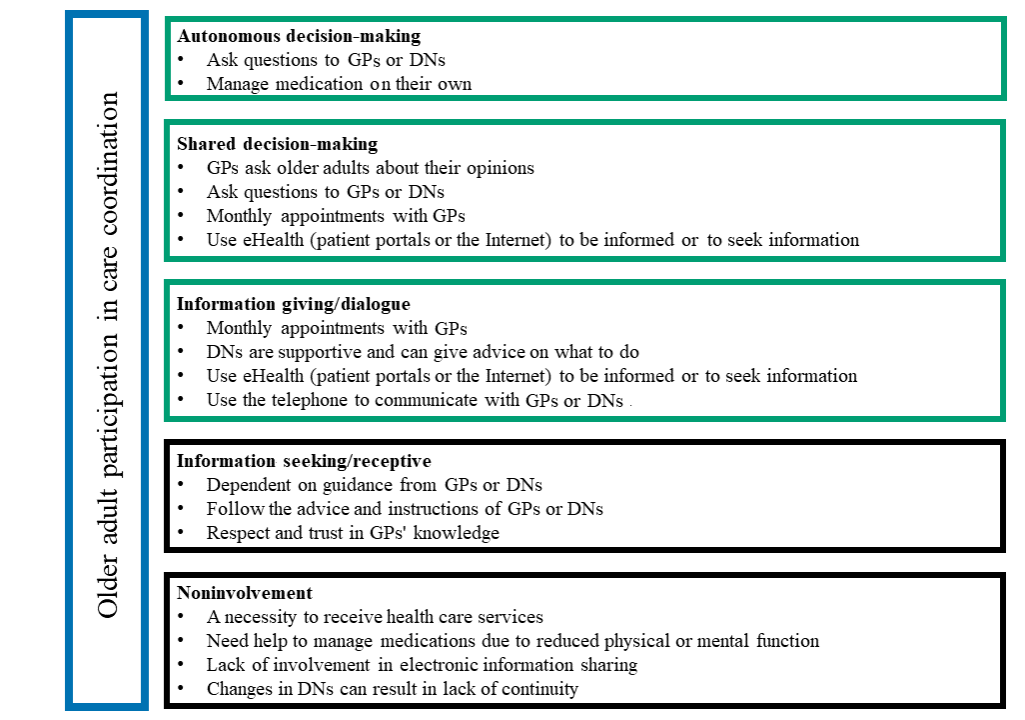
An overview of participation in care coordination among multimorbid older adults in a primary care setting. Some aspects were common for autonomous and shared decision-making, and for shared decision-making and dialogue.

There were 2 categories of older adults: (1) older adults in charge of and using eHealth to coordinate their care, and (2) older adults with a loss of control to coordinate their care [16]. By using the 5 levels of involvement by Thompson [16], we systematized the results impacting participation in care coordination for older adults (Figure 1). In Figure 1, the first category has been visualized in green text boxes as higher levels of patient involvement [16]. Older adults who manage medication and ask questions to their GPs or DNs can achieve autonomous decision-making. Shared decision-making is facilitated by GPs who ask patients for their opinions, older adults who use eHealth to be informed or to seek new information, and monthly appointments with GPs. Monthly visits with GPs provide an arena for dialogue, in line with level 2 of patient involvement [16], which includes the use of the telephone to communicate with GPs and DNs, and a sense that DNs are supportive. The second category has been visualized in black text boxes as lower levels of patient involvement [16]. Some older adults have a low level of participation as they follow guidance from their GPs, have high respect for the GPs’ knowledge, and need help managing their medications. Other aspects impacting a low level of participation include a lack of mental or physical function to manage one’s medications, necessitating assistance from DNs and health care services. Changes in DNs can result in a lack of continuity since it is difficult for older adults to get to know them. This can cause a low level of patient participation. In addition, a lack of involvement in electronic information sharing can hamper older adults’ participation in coordinating their care.

## Discussion

### Principal Findings

The study aimed to explore multimorbid older adults’ experiences with participation in care coordination, and how eHealth may support participation. The results suggest that older adults have various experiences with participation, communication, and collaboration with their GPs and DNs, and in the use of eHealth. It is important for older adults that GPs ask them about their own opinions and that they have DNs who are supportive of managing their health. Furthermore, managing medication and using eHealth to seek information can ensure high levels of participation in care coordination. All participants called friends, family members, GPs, or DNs, indicating that the telephone is a simple and efficient means of ensuring patient involvement. However, issues related to involving older adults in care coordination remain, as some older adults perceived that their GPs or DNs did not collaborate much and did not always know what had happened during the last hospital admission. Some participants indicated that they respected their GPs’ knowledge and followed their GPs’ or DNs’ guidance and instructions. Lacking the capacity and system support to be involved made older adults lose control in care coordination. Further, reduced physical capacity to manage medication without help or difficulty remembering all information made it challenging for older adults to participate. The results pinpoint different levels of participation of multimorbid older adults in care coordination and eHealth use. This can provide health care professionals and patients with crucial knowledge of what to be aware of or what options there are when multimorbid older adults become involved in care coordination and use eHealth successfully.

### Levels of Older Adults’ Participation in Care Coordination

Our results showed that some older adults participated in care coordination, and a strong relationship with GPs seems to facilitate a high level of involvement. A strong relationship of patients with GPs and DNs can be characterized as older adults asking questions to their GPs or DNs, having monthly appointments with their GPs, DNs being supportive, and GPs asking the older adults for their own opinions. A strong relationship of older adults with their GPs and DNs is an enabler for participation in the care coordination process [3,18,28,43]. Our results also showed that having monthly appointments with GPs provides an arena to talk and ask questions. Scholz Mellum et al [26] found that older adults with multimorbidity often had regular appointments with their GPs to manage their health. Having regular appointments with GPs can facilitate a strong relationship between GPs and patients, and increase the probability of participation in their care and health [26]. Steele Gray et al [43] identified that health care professionals need to recognize community-dwelling patients with complex chronic illnesses as whole persons by listening to them and having a strong relationship with them. A recent systematic review found that continuity of care with GPs or hospital doctors was associated with lower mortality rates [44], highlighting the necessity of having a strong relationship with GPs. Parisi et al [45] reported that high turnover among GPs reduces patients’ experiences of continuity of care and is linked to poorer services and patient health outcomes. Findings from our study indicate that DNs can have a good relationship with older adults, being perceived as supportive in coordinating care. Nilsen et al [46] reported that older adults who had recently been discharged from the hospital to home characterized some DNs as marvelous and kind, as well as supportive in coordinating their care. DNs can have a central role for older adults, which is especially important if GPs are not able to follow discharged patients’ health and care.

Our results show that some older adults are able to take their medications themselves, taking control of the medication management and thus making an autonomous decision about their care [16]. Managing medication is important in care coordination, especially in reviewing medication regimens or assessing all medications or supplements a patient takes [3]. We found that some older adults need assistance from DNs because of reduced physical capacity to manage their medications. It is unclear if this is something the patients want themselves. Older adults wish to be in control and to be as independent as possible, despite reduced functional ability [47]. Other research has found that older adults find it important to be informed and feel in control of their medications [48,49], and being informed can contribute to feeling in control and ensuring that information giving, dialogue, and shared or autonomous decision-making occurs [16]. In a qualitative study of 19 older adults with multimorbidity, Löffler et al [50] found that they often were critical about their medications. In a mixed method study of older adults with polypharmacy (prescribed five or more medications), Clyne et al [51] found contrasting views on taking medication, where one group of older adults believed strongly in the medication and another group were concerned about adverse effects. Similarly, we found that there are mixed experiences when older adults manage their medications. For those who are not able to manage their medications themselves due to reduced physical or mental capacity, DNs play an important role in supporting them. Robinson et al [52] highlighted the pivotal role of DNs in following up on medication and helping older adults to adhere to the medication regimen. However, our results show that some older adults trust themselves more than DNs when managing their medications. Similar results were reported by Schiøtz et al [53], with study participants not feeling confident that DNs had the necessary information about their medications. Schiøtz et al [53] highlighted that having a physician coordinating their care made the included patients feel secure about the care and medication they received. Our results suggest that for some older adults, DNs are supportive and assist them in managing their medications.

Our findings show that older adults with multimorbidity have different experiences engaging in their health. Some are in charge, while others have lost control of care coordination. According to the 5-level ladder of involvement by Thompson [16], noninvolvement is the lowest level, in which patients do not wish to be involved in discussions regarding their health. Our results indicate that patient preference is not the only factor. Noninvolvement can also be explained by a lack of system support, and GPs and DNs not having sufficient information about older adults’ health and therefore not supporting them. Other aspects impacting noninvolvement include reduced physical or mental capacity. However, according to Thompson’s description of being information seeking or receptive, patients trust the information given by health care professionals without really being involved [16]. Similarly, our results show that some older adults seem to respect GPs’ knowledge and advice so much that it hampers their participation. This is also reported in other studies involving older adults [26,49,54,55], especially those of advanced age [49]. Some older adults accept that decisions are made for them [54] and follow recommendations from physicians or nurses [26,55] without asking questions. Osborn et al [56] also found that health care professionals often neglect to include older adults with chronic illnesses in decision-making regarding their health. The study identified a lack of information sharing and talking with older adults about what they could do to promote their health [56]; this can act as a barrier to both care coordination and participation. Oksavik et al [57] also found various levels of participation when older adults with multimorbidity engaged in care planning meetings with health professionals. Thompson pointed out that the different levels of involvement are dynamic and that patients can move between levels [16]. For example, being too sick after a hospital admission makes it challenging for older adults to remember all information regarding their health and participate in coordinating their care. In a situation of acute illness or worsening of chronic illness, older adults move to a lower level of participation [16], impacting their capacity to manage care coordination. Health care professionals aware of this can provide extra education or advice to older adults in acute situations. Doing so can ensure that patients can easily self-manage their illnesses and navigate health care services, thus reducing health care use [9,10].

### Role of eHealth in Older Adults’ Participation and Care Coordination

Our results showed that some older adults use eHealth tools to communicate, seek information about their health by using a patient portal, find information on the internet, or send electronic messages. The study participants were able to use the telephone to communicate and be informed about their chronic conditions with GPs, DNs, or nurses at the GP office. Fjellså et al [21] reported that the telephone is one central eHealth tool that facilitates care coordination for older adults living at home. Oh and Lim [58] found that older adults who experienced negative communication with health care providers (ie, not receiving sufficient information on a health issue) started to search for information on the internet. However, those study participants did not have multimorbidity and were experienced with using the internet [58]. Some older adults in our study were able to use the internet to search for information, access their electronic patient portal, or send electronic messages to their GPs; this was an enabler for participation. These older adults had a degree of eHealth literacy, defined as “the ability to seek, find, understand, and appraise health information from electronic sources and apply the knowledge gained to addressing or solving a health problem” [59]. Previous research [21,60] has indicated that adults who are very old (ie, aged 80 years or over) have limited use of eHealth. Some of our study participants were over 80 years old and used eHealth. This may indicate that eHealth literacy can be found in individuals of advanced age. Other older adults did not use eHealth but were able to use their computers or tablets to pay bills digitally or play games. Older adults can have computer literacy without having eHealth literacy [59]. As 1 participant reported, older adults need to be educated to enable the use of eHealth. Research has shown that sufficient technical support and education are essential to increase eHealth use and health literacy among older adults [21,61-63]. Increasing eHealth literacy can ensure that older adults can use relevant information to self-manage their chronic illnesses as part of care coordination, which is an important enabler for both participation [17] and health promotion [15].

### Strengths and Limitations of the Study

Our study included 20 participants recruited from different GP practices in a Norwegian municipality, which ensured various patient characteristics and experiences in several aspects (participation, care coordination, and eHealth use) [64]. The recruitment of older adults may have been impacted by GPs’ or nurses’ busy work schedules and their subsequent capacity to recruit. They may also have perceived that the patients lacked the ability or interest to participate [65]. There was variation in what older adults said in the interviews; some did not provide long descriptions, while others gave well-articulated descriptions of their experiences. By including 20 older adults in the study, we were able to identify the variation among the participants, and this ensured that we met sufficient information power for the aims of our study [64]. The use of a convenience sampling strategy may also have led to missing experiences and opinions from older adults with extensive experience with eHealth and participation in care coordination [39]. However, we included older adults with multiple chronic conditions who were living at home and who were very sick and had excessive health care use. They can be “hard-to-reach” research participants; thus, this study addresses the needs and knowledge of a vulnerable group of older adults [66]. This knowledge will benefit both the general population and older adults with and without extensive experience with eHealth and participation in care coordination.

In this study, we included only experiences regarding health care services, not services from community social care and mental health. However, we included questions to map the sources of services, and no participant mentioned other services than health care, even though some mentioned having issues with depression or anxiety. Future research should highlight both mental health and social services for older adults living with multimorbidity.

### Relevance to Clinical Practice

Based on our results and discussion, participation in care coordination among older multimorbid adults, which supports the self-management of chronic conditions, can be improved by ensuring communication, facilitating management of one’s own medications, promoting a strong relationship with health care personnel, encouraging use of eHealth for information, and educating on eHealth tools. Health care professionals in charge of older adults’ care should attempt to map the individual’s willingness to participate in care coordination and to allow them to speak their minds. This is perhaps particularly applicable to GPs, as this study shows that GPs appear to have an important role in care coordination for older adults with multimorbidity. Many of the participants in this study wanted to manage their medication themselves, and supporting older adults to do this is important, despite any reduced physical ability. eHealth has shown promise as a tool to improve older adults’ participation in decision-making concerning treatment and care coordination [16]. However, older adults must be given the opportunity and education to use eHealth to participate in coordinating their care. The use of the telephone with GPs or DNs to share information, ask questions, and support self-management of chronic illnesses is important for older adults. Future research should increase knowledge of how health care providers support participation and use of eHealth in care coordination for older adults with multimorbidity. It is especially relevant for future research to achieve a better understanding of electronic information sharing between GPs and DNs, as this can trigger various levels of patient participation. Thus, promoting and sharing information about available eHealth tools, and electronic information sharing with patients and personnel are important.

### Conclusion

In this study, we explored older multimorbid adults’ experiences with participation in care coordination and their use of eHealth to support such participation. High levels of participation include aspects such as communication, asking questions to GPs or DNs, managing one’s medication, being asked questions by GPs, and using eHealth. Reduced physical capacity or reduced capacity to remember information and lack of system support can make it difficult to participate in all aspects of care coordination. The study findings are connected to Thompson’s 5 levels of involvement [16], providing avenues for future research, and giving practitioners and policymakers a better understanding of how to increase older adults’ participation in care coordination. Future research should contribute to a better understanding of electronic information sharing among health care providers because older adults may experience a lack of involvement in information sharing, thus hampering participation. Moreover, the results indicate that there is a willingness among older adults to use eHealth to participate in decision-making and to self-manage the coordination of their care.

## Abbreviations

AIC: acute inpatient care
DN: district nurse
GP: general practitioner
ICT: information and communication technologies
